# Factors Affecting Material-Cart Handling in the Roofing Industry: Evidence for Administrative Controls

**DOI:** 10.3390/ijerph18041510

**Published:** 2021-02-05

**Authors:** Zhenyu Zhang, Ken-Yu Lin, Jia-Hua Lin

**Affiliations:** 1 College of Built Environments, University of Washington, Seattle, WA 98105, USA; 2 Department of Construction Management, University of Washington, Seattle, WA 98195, USA; kenyulin@uw.edu; 3 Safety and Health Assessment and Research for Prevention (SHARP), Washington State Department of Labor and Industries, Olympia, WA 98501, USA; lija235@lni.wa.gov

**Keywords:** overexertion in pulling and pushing, material cart handling, roof construction, ergonomic risk factors, administrative control

## Abstract

Material-cart handling can be strenuous and lead to overexertion injuries. The aim of this study is to produce a thorough understanding of how the cart condition, tire type, physical environment-related factors, and load interact to influence the ergonomics and productivity of cart handling. Eighteen roofing carts with different conditions, tires, and loads were tested by one subject on three laboratory tracks: one L-shaped, one with ramps within constrained spaces, and one with obstacles within constrained spaces. A multiple linear regression analysis was performed to quantify the main and interaction effects of the factors of interest on the cart operations. The research findings confirm that using aged carts increases the injury risk by as much as 30.5% and decreases productivity by 35.4%. Our study also highlights the necessity of keeping an open space for cart operation; the travel distance from a cart to a ramp/obstacle should be greater than 61 cm. Finally, the results suggest the at-risk thresholds for different ramp slopes and obstacle heights, and the safe load capacities for the various working circumstances that are common on construction sites. The evidence created in this study can be translated into administrative controls for cart handling to reduce overexertion injuries and enhance performance.

## 1. Introduction

Manual carts are some of the most common material-handling equipment in various industrial settings. Cart maneuvering can be strenuous and may result in overexertion in pulling and pushing (OPP), a leading cause of work-related musculoskeletal disorders (WMSDs). OPP caused approximately 385,000 injuries from 2011 to 2017, constituting 15% of the WMSDs in the United States [1]. Similarly, in the U.S. construction industry, OPP accounted for 11.2% of the total lost days caused by WMSDs [2].

Extensive scholarly attention has been given to the ergonomic design of manual carts. The studied design parameters include the handle position [3,4], handle height [5,6], handle orientation and friction [7], inter-handle distance [8], and wheel diameter [5,9,10]. Although these engineering controls can prevent cart operators from encountering hazardous working conditions, it is widely accepted that administrative controls, namely, management initiatives that modify work procedures and processes [11], are needed as complementary measures to develop comprehensive and successful workplace interventions [12,13,14,15].

Despite their acknowledged importance, administrative controls have not been an explicit subject of ergonomics research, with little empirical evidence being offered to support the design of these critical measures. For example, cart maintenance and replacement are warranted to avoid overexertion and productivity loss caused by cart deterioration [16,17,18]. Although these studies acknowledged the detrimental effect of cart deterioration, only subjective and descriptive narratives were provided without any numerical evidence. Little is known about the degree to which a cart’s ergonomics and productivity performance will deteriorate over time, giving practitioners limited evidence to determine whether preventive maintenance is a worthy decision and when is the best time to proceed with it.

The tire type affects the cart operation. Solid (polyurethane foam–filled rubber) tires have been recently introduced in the construction industry as a replacement for pneumatic (air-filled rubber) tires. A solid tire will not become flat or deformed; however, it is heavier and more expensive. To our knowledge, no study has been dedicated to empirically comparing these two tires when incident prevention and productivity enhancement are prioritized.

Workplace-layout planning is an administrative control to reduce physical environment-related hazards for cart handling. The adverse effects of physical environment-related factors have been confirmed; however, the research efforts thus far have been focused on addressing single hazards, e.g., obstacles [19,20], ramps [21,22], or space constraints [6,23]. For example, Nimbarte et al. [22] measured the effects of three ramp slopes (0, 5, and 10 degrees) on the musculoskeletal loads of cart operators. At workplaces, however, it is not uncommon to see physical environment-related factors at play simultaneously, such as moving a cart up a slope within a tight space. To competently maneuver in workplace layouts, practitioners should be aware of the effects of multiple hazards, rather than single hazards.

This is particularly critical for the construction industry, where work stations are constantly evolving as projects progress, adding substantial variability to the physical environment-related factors [24,25,26]. Another limitation of existing studies is that the effects of physical environment-related factors are primarily measured at low cart-load ranges: from 20 to 170 kg. It remains unknown whether the findings apply to higher cart-load ranges [6], e.g., 243 to 469 kg, which are commonly seen on construction sites.

Finally, the cart load is the foremost risk factor for OPP [10,27], and workers should refrain from overloading a cart by following load restrictions. Several efforts have been made to provide such numerical standards [28,29,30]. However, none of them consider the impacts of the cart condition, tire type, and physical environment-related factors; only fixed thresholds are recommended which are not adaptable and thereby limit their applicability when imposing load restrictions under various working conditions.

The objective of this study is to measure how cart condition, tire type, physical environment-related factors, and load interact to influence cart operations to support the design of administrative controls for cart handling. In Experiment 1, we measured the forces and time required to operate carts of different conditions and equipped with different tires during dynamic cart pulling in straight-line and turning motions. Overexertion risks were estimated to offer empirical advice for cart maintenance, replacement, and tire selection. In Experiment 2, we focused on cart operations in relation to a combination of physical environment-related factors and loads to provide advice for workplace layout and load restrictions.

Our study rests on the premise that, to create transferrable evidence that practitioners can immediately adopt, laboratory experiments should acknowledge practical complexity and uncover the effects of risk factors in detail, by closely simulating actual working situations [31]. Owing to the authors’ participation in an ergonomics program with a large roofing contractor in the U.S., our experiments managed to properly determine the levels and interactions of the risk factors of interest. Hence, our research findings apply strongly to applications in the roofing industry, or other industrial settings where similar scenarios exist, to reduce overexertion and enhance cart-handling performance.

## 2. Experiment 1

The objective of Experiment 1 was to uncover the effects of the cart condition and tire type on the ergonomics and productivity of cart operations under various loads.

### 2.1. Materials and Methods

#### 2.1.1. Experiment Design

##### Apparatus

**Figure 1:**
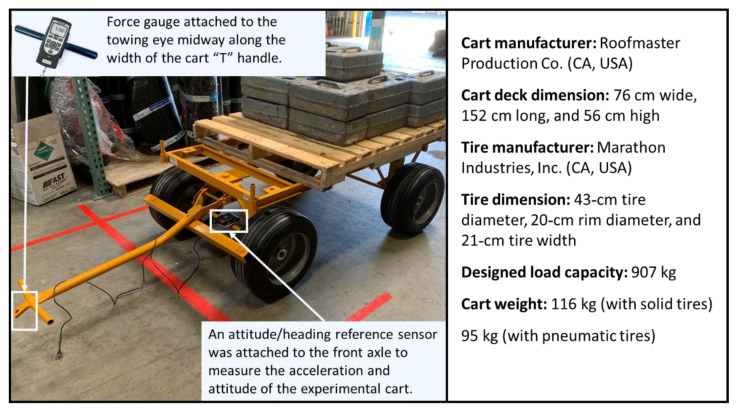
Instrumented cart.

A typical cart in the roofing industry is composed of a deck (for loading materials), a turntable (in front of the deck), and a height-adjustable “T”-shaped handle (connected to the turntable for pulling) (Figure 1). In this study, 18 carts of this type with varying ages and tire types were tested; newer carts were generally in better condition (Table 1). The framing of the five-year-old carts was 10 kg lighter than that of the newer carts. To make the results comparable, an extra 10-kg load was added to the five-year-old carts during the experiment. Both pneumatic and solid tires were from the same manufacturer and had the same dimensions.

A digital force gauge (Series: DFS2, John Chatillon & Sons Co., Largo, FL, USA) with a maximum reading of 1000 N and a precision level of 0.0001 N was used to measure the required operating forces (ROFs) for cart maneuvering. As shown in Figure 1, the force gauge was attached to the towing eye midway along the width of the handle, and the force gauge was perpendicular to the handle while pulling. The force data were transmitted telemetrically to a personal computer at 36 Hz.

An industrial-grade attitude/heading reference sensor (Series: 3DM-CV5-25, Lord Sensing Co., Williston, VT, USA) was mounted on the cart’s front axle to gather acceleration and attitude data at 50 Hz to characterize the cart movement and to measure the required operating time (ROT). The positioning consistently oriented the *x*-axis vector of the sensor frame with the forward direction of the cart movement, and the *z*-axis vector was perpendicular to the cart frame. The resolutions for acceleration and attitude data were 0.05 mg and 0.003°/s, respectively.

##### Laboratory Track

**Figure 2:**
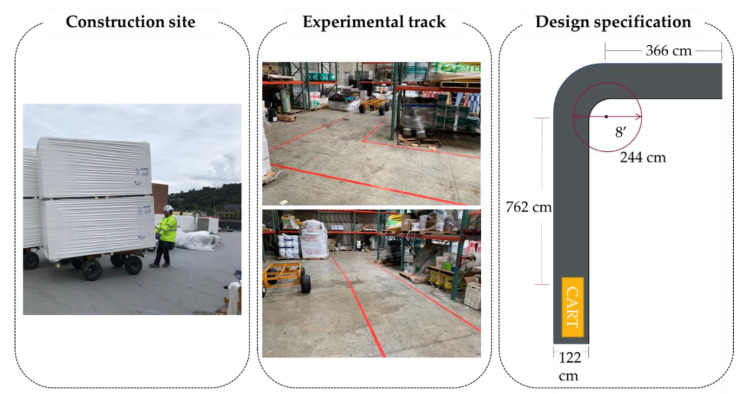
L-shaped track.

An L-shaped track (Figure 2) was constructed to represent the common working conditions for cart operations. The track allows dynamic pulling activities in both straight-line (762 cm long) and turning motions (244-cm diameter). Tapes were used to mark a 122-cm-wide lane for cart operation.

##### Subject

Individual differences are a known factor in OPP [23,32,33,34,35]. We controlled for individual factors by using a single subject. A male graduate student, aged 29 years, with a stature of 177 cm and a body mass of 88 kg, participated in the experiment. The anthropometric characteristics of the subject are representative of construction workers in the greater Seattle metropolitan area, based on a regional worker-profile survey [36]. The subject is healthy and free from any musculoskeletal problems. The subject visited five roofing jobsites to videotape and learn how workers operated carts. Then, he received 30 min of training on pulling techniques from two experienced workers and was given observational feedback in practice trials to ensure that identical pulling techniques were applied.

##### Experimental Procedure

The subject was instructed to pull a cart forward for 762 cm and then make a 90° turn with a 244-cm diameter, followed by a 366-cm straight pull to the marked finish point. The cart operations were performed from and until a standstill. The participant mimicked the pulling techniques of the roofers by pulling the cart symmetrically with both hands and without jerking motions. The handle was at the thigh level and the participant bent his back slightly (less than 15°) when initiating and sustaining the cart movement. The cart-movement speed was subjectively controlled by the participant at an approximate walking speed of 2 m/s.

All of the trails were performed with three cart loads: 243, 356, and 469 kg, by combining a wooden pallet weighing 16 kg with 20 ballast weights, each weighing 22.7 kg. The ballast weights were loaded evenly onto the wooden pallet, which was placed on an experimental cart. All pneumatic tires were inflated to the recommended pressure. Good housekeeping was performed to ensure the track was free from debris and congestion. Each experimental setup was repeated at least three times until consistent ROF measurements were obtained: each measurement was within 15% of the others. A one-minute recovery time was given between trials and a three-minute rest was given between experimental setups to eliminate fatigue [23,37,38]. All 162 experimental trials (18 carts × 3 cart loads × 3 trials) were completed in five days.

#### 2.1.2. Data Analysis

##### Data Management

**Figure 3:**
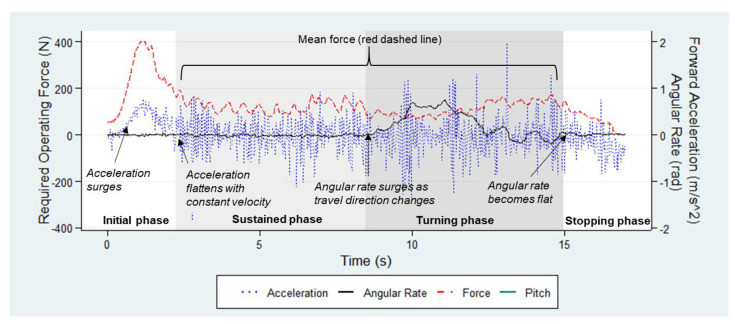
Sample trial with the L-shaped track.

The cart movement on the L-shaped track was divided into four phases: initial, sustained, turning, and stopping (Figure 3). The four motion phases were determined post hoc using forward-acceleration and angular-rate data collected by the attitude/heading reference sensor. The “Segmented” package in the R project [39] was employed to detect the breakpoints where the linear relations between the acceleration/angular rate and time changed (Figure 3). The mean force over the sustained and turning phases, which constituted the majority of the cart movement, was obtained as the primary outcome measure of the ROF for statistical analysis. The required operating time (ROT) to finish the course of motion on the track was recorded as a measure of productivity.

##### Statistical Analysis

Experiment 1 employed two full-factorial designs. In Experiment 1a, we included three dummy independent variables for the cart condition to compare a one-year-old cart, five-year-old cart, and five-year-old cart after tire replacement against a brand-new cart (the reference category) and a continuous independent-variable cart load. Two multiple linear regression models were created to investigate the effects of the cart condition on the ROF and ROT under different cart loads. Because the five-year-old experimental carts (donated by our industry partner) were all equipped with solid tires, the tire type was controlled in Experiment 1a by using only carts (Table 1) that were equipped with solid tires.

In Experiment 1b, we included a binary independent-variable tire type (pneumatic and solid tires) and a continuous independent-variable cart load to estimate the association between the tire type and the ROF/ROT under different loads. The cart conditions (brand-new and one-year-old) were also included in the models as a precision variable to increase the precision of the estimate.

To understand how the changing ROFs are associated with the OPP risk, the ROFs were converted into capability percentages (CPs) by referencing the Snook Table [40]. Because cart operations are mostly performed by male workers in the construction industry, the CP denotes the percentage of the male population who are psychophysically capable of performing the experimental pulling task. The parameters needed for the Snook Table when calculating CPs included pulling at thigh height, 46 m pulling distance, and one pull every five min, which were determined through field observations. A multiple regression analysis was also performed for the CP.

For both ROF and CP, we reported regression coefficients (β) to present the estimated change in the absolute value corresponding to the unit change in the independent variable, along with the robust standard error (s.e.) and *p*-value. The ROT was log-transformed to compare the relative percentage change in productivity. Backward elimination was applied to include interaction terms between the independent variables. All possible interactions were first fitted together to screen for significance, and only the terms that were significant at a level of 0.05 were included in the final models. All analyses were performed using the R Project software.

### 2.2. Results

**Figure 4:**
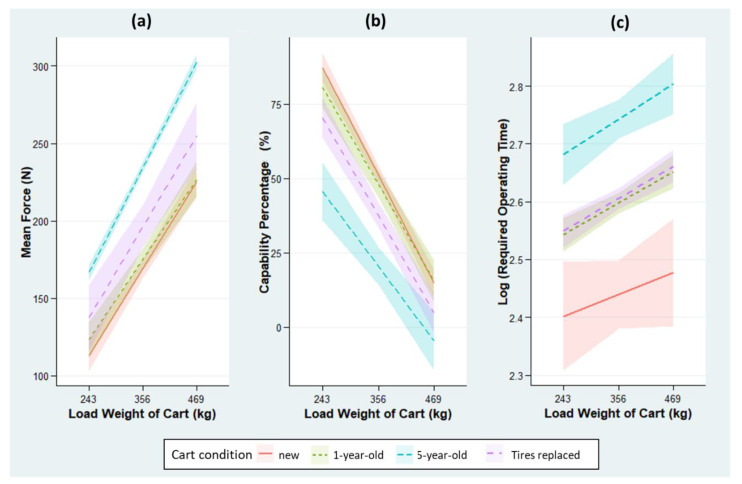
Estimated values: (**a**) ROF, (**b**) CP, and (**c**) ROT for carts of different conditions by fitting the actual measurements in Experiment 1a from the final linear regression models. The ribbons indicate 95% confidence intervals.

A total of 99 trials were conducted for Experiment 1a to examine the effects of the cart conditions on the cart operation under different loads. Table 2 summarizes the regression analysis results with the observed β, s.e., and *p*-value for the three dependent variables: ROF, CP, and ROT. The R-squared values for the three models are 0.83, 0.81, and 0.62, indicating the goodness-of-fit of the regression models. The estimated ROF, CP, and ROT for carts of different conditions are shown in Figure 4.

According to Table 2, both the cart condition and load are significantly associated with the ROF and corresponding CP, in most cases. A difference of 65.5 N in ROF (30.6% in CP) was found between the new and five-year-old carts, despite no significant difference between the new and one-year-old carts. Replacing the old tires on the five-year-old carts was effective in lowering the ROF (additional forces decrease from 65.5 to 27.1 N) with a moderate margin in CP (from −30.6% to −13.4%). In addition, the ROF increased as a cart was more heavily loaded, with an incremental increase of 0.487 N for every 1 kg increase in cart load, on average, for carts of different conditions.

Table 2 also shows that the cart condition and load are significantly associated with the ROT. The productivity of the cart operation degrades as the carts age: one- and five-year-old carts respectively require 17.1% and 35.4% more time to complete each trial, on average, than a new cart. Tire replacement on a five-year-old cart has proven effective in increasing the productivity (the extra percentage change in ROT decreases from 35.4% to 18.0%). Lastly, the ROT increases by 0.046% for every 1-kg increase in the cart load, on average, for carts of different conditions.

**Figure 5:**
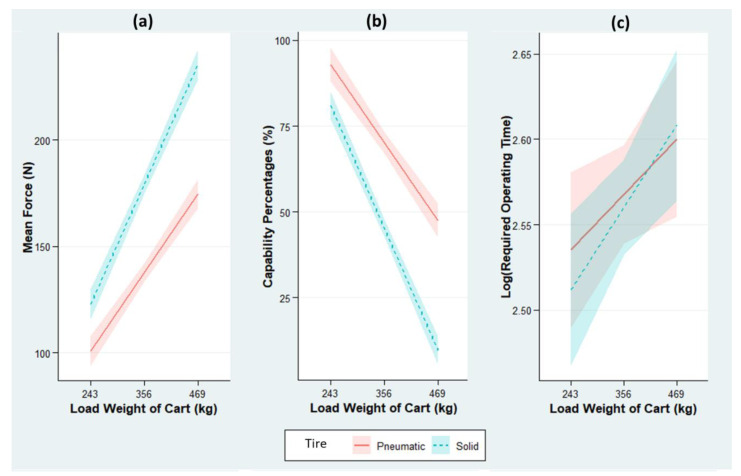
Estimated values: (**a**) ROF, (**b**) CP, and (**c**) ROT for two tire types by fitting the actual measurements in Experiment 1b from the final linear regression models. The ribbons indicate 95% confidence intervals.

A total of 126 trials were conducted for Experiment 1b to compare the difference between pneumatic and solid tires. The regression-analysis results are summarized in Table 3 and displayed in Figure 5. The R-squared values for the three models are 0.88, 0.87, and 0.40, indicating the goodness-of-fit of the regression models. The tire type is significantly associated with the ROF and CP; however, the measures of the association are dependent on the cart load, as we observe a significant interaction effect between the tire type and load. The intercept values estimate that pulling a 243-kg brand-new cart equipped with pneumatic tires requires 92.6 N, which is acceptable for 98.1% of the male population.

At this baseline cart load (243 kg), solid tires are estimated to require 22.0 N more force than the pneumatic tires, which is equivalent to an 11.9% decrease in CP. As the carts are more heavily loaded, however, the increase in ROF and decrease in CP become more rapid among carts with solid tires (Figure 5a,b). With every 1-kg load added to a cart, the ROT is estimated to increase 0.326 N (−0.201% in CP) for pneumatic tires, while solid tires require an additional 0.173 N of force and impose a 0.115% extra risk (i.e., −0.115% in CP) over pneumatic tires. Finally, Table 3 shows that the productivity of the cart operation is not significantly associated with the tire type (*p*-value = 0.645).

## 3. Experiment 2

The objective of Experiment 2 was to uncover the main and interaction effects of physical environment-related factors (obstacle height, ramp slope, and space constraint) on the cart operation under different loads.

### 3.1. Materials and Methods

#### 3.1.1. Experiment Design

##### Apparatus

Four brand-new carts (two with pneumatic tires and two with solid tires) were tested in Experiment 2. The other deployed apparatuses were identical to those in Experiment 1.

##### Laboratory Track

**Figure 6:**
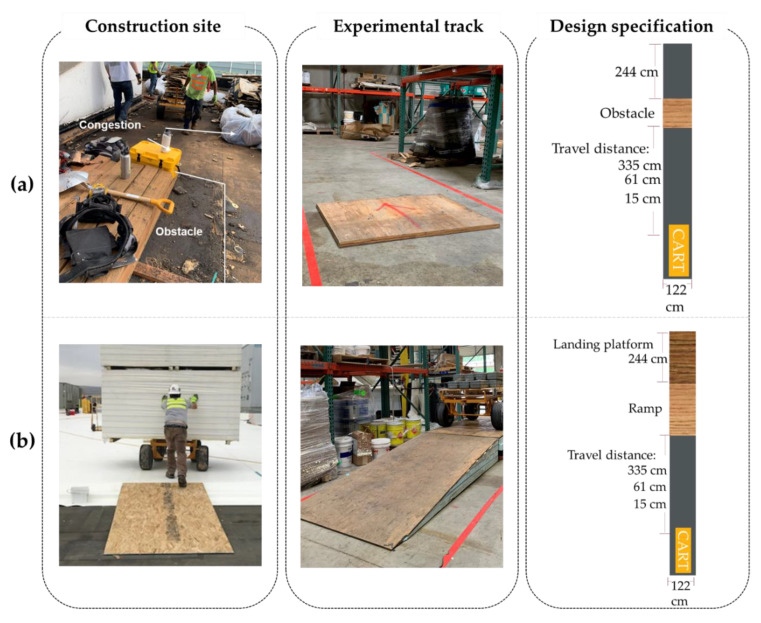
Laboratory tracks for Experiment 2: (**a**) obstacle track and (**b**) ramp track.

Two laboratory tracks were created by the authors and two roofers to simulate the actual physical environments on construction sites. First, an obstacle track (Figure 6a) was arranged by mounting a 122 cm × 122 cm plywood board using anti-slip traction tape. The plywood boards came in three heights: 1.9, 3.8, and 5.7 cm. Second, a ramp track (Figure 6b) was built with two 244 cm × 122 cm plywood boards attached to insulation boards. The ramp came in three slope gradients: 4°, 8°, and 12°.

Three levels of space constraint were simulated by defining the travel distance (i.e., from the front axle of the cart to the closest edge of an obstacle/ramp): 335, 61, and 15 cm. The 335-cm travel distance simulated maneuvering a cart within an open space, which allowed the cart to build momentum and sustain a relatively constant velocity before contacting a ramp/obstacle. The 61-cm distance simulated a moderately constrained space and was determined by measuring the actual travel distances in congested passages, corners, and construction man-lifts. The worst scenario was 15 cm, when the front wheels almost contacted the edge of the ramp/obstacle, providing no space to generate momentum. All levels and interactions of the risk factors of interest were determined by 10 field observations and surveying 25 field workers in order to consider all possible working environments for cart operations.

##### Subject

The same as in Experiment 1.

##### Experimental Procedure

A cart was first placed upon a level surface with an obstacle/ramp located at a pre-defined distance (355, 61, or 15 cm) to simulate space constraints. The subject was requested to follow a set procedure: pull the cart over the pre-defined distance, then surmount the obstacle/ramp, followed by a 244-cm straight pull to a marked finish point. The cart operations were performed from and until a standstill. The entire experiment was conducted with five loads: 16, 129, 243, 356, and 469 kg. The subject mimicked the roofers’ pulling technique by jerking to overcome an obstacle/ramp when within a moderately (61-cm travel distance) or extremely (15 cm) constrained space. The other experimental details were identical to those in Experiment 1. All 963 experimental trials were completed in 25 days.

#### 3.1.2. Data Analysis

##### Data Management

**Figure 7:**
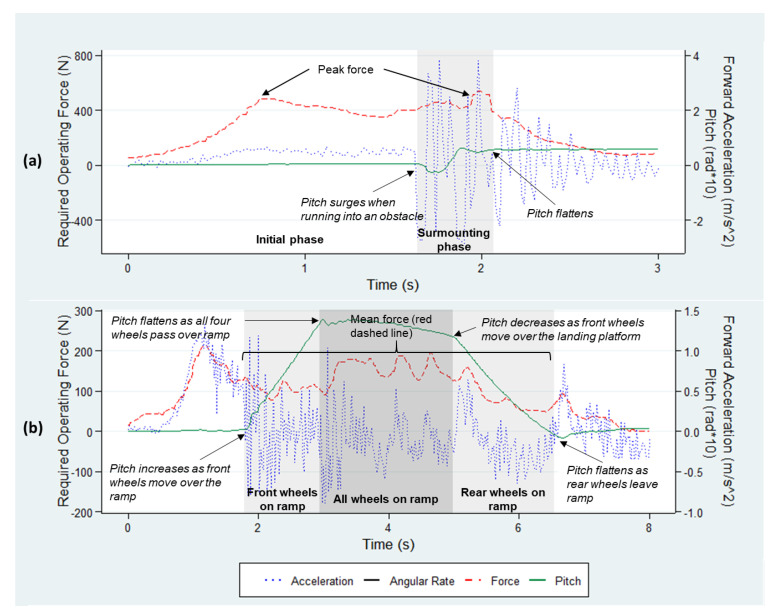
Sample trial data: (**a**) obstacle track within a moderately constrained space and (**b**) ramp track within a severely constrained space.

The cart movement on the obstacle track was divided into initial and surmounting phases (Figure 7a). The two phases were determined post hoc using pitch data, which denote the angle between the longitudinal axis of the cart and the horizon. As surmounting an obstacle requires impulsive forces, the peak force during the initial and surmounting phases was recorded as the ROF. The duration of the initial and surmounting phases was collected as the ROT.

The cart movement on the ramp track was divided into three phases: front wheels on ramp, all wheels on ramp, and rear wheels on ramp. Similarly, the three phases were defined by detecting the change in the pitch with time (Figure 7b). The mean force and operating time over the three phases were recorded as the primary outcome measures for ROF and ROT, respectively.

##### Statistical Analysis

Experiment 2 employed respective full-factorial designs for the obstacle and ramp tracks. As an investigation of the effects of the obstacle height and space constraint on the cart operations under different cart loads, Experiment 2a included a continuous independent-variable obstacle height, two dummy independent variables for space constraints to compare moderate space constraints and extreme space constraints against *open space* (the reference category), and a continuous independent-variable cart load.

Experiment 2b was designed for the ramp track and included a continuous independent-variable ramp slope, two dummy independent variables for space constraint, and a continuous independent variable cart load. The tire type was included in both Experiments 2a and 2b as a precision variable. The parameters needed for estimating the CP were pulling at thigh height, 7.6-m pulling distance, and one pull every five min. The other technical details for data analysis replicated those of Experiment 1.

### 3.2. Results

Owing to the capacity constraint of the force gauge, 13 setups for Experiment 2a (obstacle track) were not tested. The remaining setups were tested over 501 trials. Table 4 summarizes the regression analysis results with the observed β, s.e., and *p*-value for the effects of the obstacle height, space constraint, and cart load on the ROF, CP, and ROT. The R-square values for the three models are 0.92, 0.89, and 0.96, indicating the goodness-of-fit of the regression models.

First, the obstacle height was found to be significantly associated with the ROF when overcoming an obstacle within an open space: The ROF is estimated to increase by 25.0 N for every 1-cm increase in obstacle height; however, the additional ROF does not lead to a significant difference in CP (*p*-value = 0.057). Second, Table 4 confirms the effect of the space constraint on the ROF and CP in the baseline scenario: when overcoming a 1.9-cm obstacle with a 16-kg cart, the ROF is estimated to increase by 46.8 N (insignificant difference in CP; *p*-value = 0.772) and 190 N (45% decrease in CP, *p*-value < 0.001) in moderately and extremely constrained spaces, respectively, as opposed to an open space. Third, the ROF increases by 0.546 N with every 1-kg load added to a cart when overcoming an obstacle within an open space.

**Figure 8:**
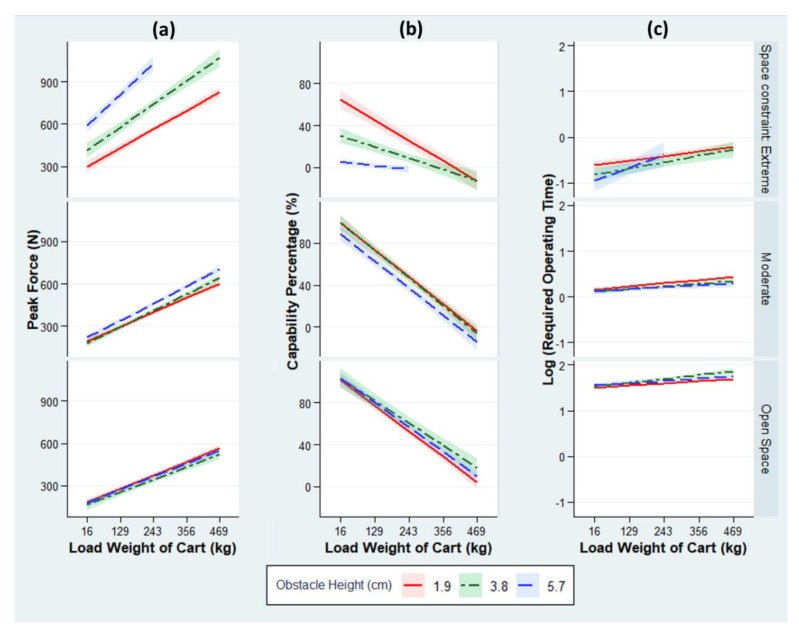
Estimated values: (**a**) ROF, (**b**) CP, and (**c**) ROT for three obstacle heights by fitting the actual measurements in Experiment 2a from the final linear regression models, stratified by the space constraint. The estimated values are adjusted for tire type. The ribbons indicate 95% confidence intervals.

Fourth, the effect of the obstacle height was found to differ under different levels of space constraint, as we observed a significant interaction effect between the obstacle height and the space constraint. Specifically, the adverse effects of the obstacle height on the ROF and CP became significantly salient in an extremely constrained space (Figure 8a,b). According to Table 4, a higher obstacle (by every 1 cm) requires 76.8 N of additional force and imposes an extra 14.2% risk in an extremely constrained space, when compared to an open space.

In contrast, the adverse effect of the obstacle height on the ROF was not significantly stronger in a moderately constrained space than in an open space (*p*-value = 0.569), while a significant but minimal gap in CP (−3.77%) was found. Furthermore, the effect of the space constraint was found to differ under different levels of cart load. As the carts were more heavily loaded, the changes in ROF/CP became more rapid in a constrained space (Figure 8a,b). For every 1-kg increase in cart load, the moderate and extreme space constraints required an extra 0.386 N of force (−0.028% in CP) and 0.845 N of force (0.072% in CP), respectively, when compared to an open space.

Finally, Table 4 suggests that the productivity of the cart operation is not significantly associated with the obstacle height (*p*-value = 0.204). The ROTs under different space constraints simply reflect travel distances and are not worthy of discussion.

**Figure 9:**
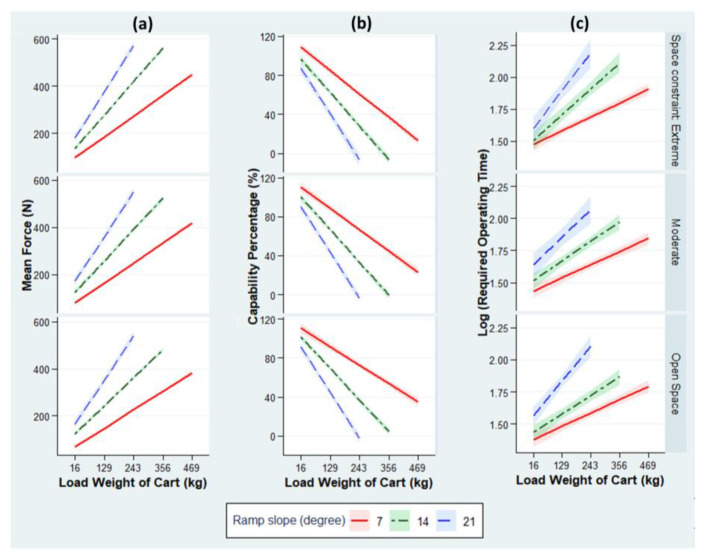
Estimated values: (**a**) ROF, (**b**) CP, and (**c**) ROT for three ramp slopes by fitting the actual measurements of Experiment 2b from the final linear regression models, stratified by the space constraint. Estimated values are adjusted for tire type. The ribbons indicate 95% confidence intervals.

Owing to the capacity constraint of the force gauge, 26 setups for Experiment 2b (ramp track) were not tested. The remaining setups were tested over 462 trials. The regression analysis results are summarized in Table 5. The R-square values for the three models are 0.91, 0.89, and 0.50, indicating the goodness-of-fit of the regression models. The ramp slope is significantly associated with the ROF and CP; however, the measures of the association depend on the cart load, as we observe a significant interaction effect between the ramp slope and cart load. When overcoming a 16-kg cart, the ROF is estimated to increase by 10.3 N (−2.29% in CP) for every 1° increase in ramp slope. However, the detrimental effect of the ramp slope significantly escalates as the carts are more heavily loaded (Figure 9a,b). Table 5 shows that, with every 1-kg load added to a cart, the ROF is predicted to increase by 0.756 N (−0.196% in CP) for a 4° ramp, while a steeper ramp (for every 1°) requires 0.123 N of additional force and imposes an extra 0.03% risk.

Table 5 also confirms the effect of the space constraints on the ROF and CP. After adjusting for other factors, the ROF is estimated to increase by 19.2 N (−3.92% in CP) and 41.5 N (−8.76% in CP) under moderately and extremely constrained spaces, respectively, when compared to an open space.

Finally, the impact of the ramp slope on the ROT is statistically significant, while the magnitude of the impact varies under different loads (Figure 9c). For the baseline cart load (16 kg), the ROT to overcome a ramp is 2.19% longer for every 1° increase in slope; however, the ROT gap between two ramps differing in slope by 1° grows increasingly wider as the carts are more heavily loaded (by 0.015% for every 1-kg increase in cart load).

## 4. Discussion

This study assessed the main and interaction effects of the cart condition, tire type, physical environment-related factors, and load on the cart operation, using the roofing industry as the study context. Evidence was collected to uncover the ergonomic hazards that should be addressed to prevent overexertion injuries resulting from cart handling.

### 4.1. Effect of Cart Condition

The results of Experiment 1a made it clear that operating a five-year-old cart was risky (placing an additional 30.5% of the male population at an elevated risk for overexertion) and non-productive (requiring 35.4% extra time to complete the experimental task). The malfunctions of old tires (contaminated tires and broken/deformed bearings) appear to explain 50% to 59% of the performance degradation, as we observed a 17.2% increase in CP and 17.4% decrease in ROT after tire replacements for five-year-old carts. Altogether, the findings suggested that tire replacement is a convenient administrative control worth considering; however, it is less effective than replacing the entire cart, when structural framing problems (warped axles and loose turntable) have developed causing excessive friction in axle shafts and vibration in the turntable.

A comparison between the results of Experiments 1a and 1b suggested that pneumatic and solid tires could have different rates of wear and tear. In Experiment 1a, we only tested carts with solid tires and found insignificant differences in CP between brand-new and one-year-old carts (Table 2). However, after including carts with pneumatic tires, we found in Experiment 1b (Table 3) that one-year-old carts presented a 7.3% higher injury risk than brand-new carts did, on average, over the two types of tire (*p*-value < 0.001). This finding implies that pneumatic tires could deteriorate more rapidly and thereby need more attention in routine maintenance.

Compared to the narrative descriptions of the adverse effects of cart deterioration in prior literature [16,17,18], our numerical evidence can be more powerful when persuading practitioners to replace aged equipment in a timely manner [41,42]. The evidence is also more business oriented by providing important data (i.e., changes in health outcome and productivity) for practitioners to perform cost-effectiveness analyses of preventative replacement. We envision the evidence as providing broader implications for roofing and other industries in which heavy-duty carts are utilized and deteriorate fairly quickly.

### 4.2. Effect of Tire Type

We identified the differences between pneumatic and solid tires, which were overlooked by prior studies. The pneumatic tires consistently outperformed the solid ones across all experimental conditions with a moderate margin in CP (L-shaped track: −11.9%, obstacle: −14.7%, and ramp: −6.96%) and a minimal margin in ROT (L-shaped track: insignificant, obstacle: 2.38%, and ramp: 4.24%), which could be ascribed to the light weight of the pneumatic tires. The larger difference in ROFs on the obstacle track could be attributed to the fact that a pneumatic tire has a greater resilience allowing it to better conform around obstacles.

Furthermore, Experiment 1b identified an interaction effect between the tire type and cart load on the ROF/CP, implying that the advantages of pneumatic tires become increasingly salient under a higher cart load. Our results supported the use of pneumatic tires, especially when transporting heavier loads. However, it should be noted that the advantages of pneumatic tires are modest, and can be offset by the labor and administrative costs caused by flat-tire downtimes.

### 4.3. Effect of Physical Environment-Related Factors

When it comes to physical environment-related factors, this study was unique and did not simply replicate the past works performed in the contexts of healthcare, waste collection, aviation, and manufacturing [19,20,21,22]. We carefully varied the degree of each physical environment-related factor and the interactions among factors to mimic the actual working conditions at construction sites. Retrospectively, we found this experiment design to be critical in generating results that could reliably inform practice.

Previous studies have not examined whether a space constraint increases the force required to overcome an obstacle/ramp, although its detrimental impact has been confirmed when moving carts along a straight line or making a turn [23,27]. In this study, Experiment 2a surprisingly demonstrated that the space constraint was the strongest risk factor when surmounting obstacles. This factor also considerably amplified the adverse effects of both obstacle height and cart load on the cart operation.

In an open space, a larger obstacle barely presented any additional risk under the different cart loads (Figure 8) because the open space allowed the operator to build sufficient cart momentum to overcome an obstacle. In a constrained space, however, the operator could not depend on the cart’s inertia of motion; rather, a much greater force is needed to not only overcome the obstacle, but also initiate the cart movement.

Our findings indicated that any evidence that overlooks space constraints as a risk factor will substantially underestimate the injury risk of overcoming an obstacle and, therefore, is unreliable when informing workspace-layout planning. Practitioners are recommended to reserve an open space in the work station to ensure that the travel distance from a cart to any obstacle is larger than 61 cm.

Experiment 2b highlighted the ramp slope as the strongest predictor of ROF, CP, and ROT when overcoming a ramp, suggesting that practitioners should build gentler ramps for cart operations. Furthermore, the detrimental effect of the ramp slope was stronger for carts with heavier loads. This could be due to the heavier carts lose momentum more easily on steeper ramps and operators need to constantly apply a much larger force to initiate the cart movement [23]. This finding unveiled a complex interaction effect between the ramp slope and cart load on the cart operation, challenging the prior observation [43] that the ramp slope has a simple linear association with the ROF. This means that past research findings [19,20] that were obtained at lower cart loads (20–100 kg) can underestimate the risk of injury when overcoming a ramp at higher cart loads. Overall, our study results support the notion that experimental studies should sufficiently consider the levels of variables and their interaction effects to provide insights into the mechanics of cart operation [6] and generate reliable evidence for applications [31].

### 4.4. Effect of Cart Load

Although the cart load has been studied by almost all the above-mentioned literature, this research contributed to the evidential understanding of this risk factor by assessing its effects at a higher range and thoroughly exploring how its effects vary in relation to the cart condition, tire type, and physical environment-related factors. Hence, the research results have practical implications for operating heavy-duty carts in dynamic and complex working environments. Practitioners can refer to Figure 4, Figure 5, Figure 8, and Figure 9 to quickly search for the safe load under a certain circumstance or to find the at-risk threshold of a physical environment-related factor, given a fixed cart load. In general, cart load has a greater effect on CP (L-shaped track: −0.289%, obstacle: −0.210%, and ramp: −0.196% for every 1-kg increase in cart load). Despite being statistically significant, the effect of cart load on ROT is rather minimal (L-shaped track: 0.046%, obstacle: 0.071%, and ramp: 0.98%).

Moreover, we confirmed that the cart load can modify the effects of the tire type, ramp slope, obstacle height, and space constraint, contradicting previous observations that the cart load is linearly proportional to the operating forces [10] and physiological demands [43].

### 4.5. Limitations and Future Directions

Similar to many previous studies [5,23,44], our practical implications were made, based on the psychophysical limits of the measured hand forces. Although the validity of the psychophysics-based Snook Table has been repeatedly confirmed after its inception [16,45,46], we still see the potential of using advanced instruments (e.g., surface electromyography) and biomechanical analyses to provide more accurate results for risk assessment. For example, an increase in the cart load or ramp slope not only required a larger hand force, but also prolonged the exertion duration of the cart operation; however, we only considered the force when assessing the OPP risk because of the restrictions of the Snook Table. Furthermore, the pulling/pushing speed has been found to be a factor that can influence the shear forces and moments within the lumbar spine [6,47]. Without considering this factor, our study could underestimate the OPP risk, especially for experimental conditions in which a constrained space was involved that required jerking motions.

The generalizability of the research findings may be limited because we controlled the individual factors in the experiment by selecting an anthropometrically representative subject who adopted the most common pulling/pushing techniques. Another way to control individual differences is to deliberately select subjects and then adjust individual factors in the statistical analysis. But thus far, the causal mechanisms of individual factors on OPP remain unclear, refraining us from applying this method. Alternatively, a large subject pool could be obtained for the full randomization of individual factors, which, however, overcomplicates and distracts the experiment design in which the factors of interest are already highly varied. This methodological choice is also constrained by the cost and difficulty of recruiting field workers.

Because of the research subject limitation, our results only represented the best estimates; the actual effects of the factors we tested may vary from one person to another. Starting from the premise that administrative controls are more effective and attainable than individual changes during a workplace intervention, this study focused on the cart condition, tire type, physical environment-related factors, and load, rather than individual factors.

Future research can certainly be built on this study to characterize and assess how individual differences might influence the OPP risk. For example, what is the best pushing/pulling technique that cart operators should use to negotiate with physical environment-related hazards? Some findings on pulling/pushing techniques can be found in the literature [23,27,34,35]; however, they are far from being conclusive.

## 5. Conclusions

Overexertion in pulling and pushing constitutes more than 11% of work-related musculoskeletal disorders in the construction industry. Through a series of laboratory experiments, this study enhanced the evidential understanding of how the cart condition, tire type, physical environment-related factors, and load could interact to influence the overexertion risk and productivity during cart handling. Specifically, we confirmed that using aged carts increases the injury risk by 30.5% and decreases the productivity by 35.4%. Also, pneumatic tires consistently outperform solid ones in both ergonomic and productivity performance. Our study further identified and quantified the interaction effects between tire type and cart load, between space constraint and cart load, between obstacle height and space constraint, and finally between ramp slope and cart load. Based on our enhanced understanding, we propose a set of principles for administrative controls (cart maintenance and preventative replacement, tire selection, workplace-layout planning, and load restrictions) that could be tied to the operational context in the roofing industry. For one, we suggest the roofing companies to pay more attention to the routine maintenance of aged carts, especially those with pneumatic tires. On the other hand, we recommend field workers to build gentle ramps, reduce obstacles, and keep an open space for cart handling. Our research results can be readily incorporated into a company’s training materials, job-hazard analysis, job-specific safety plan, and safety-inspection checklist. We envision this study as increasing practitioners’ awareness and understanding of how to ergonomically operate carts, thereby reducing overexertion injuries and their economic burden on our society. Future research could consider using advanced instruments and biomechanical analyses to examine the individual differences in cart operations and provide more accurate results for risk assessment.

## Figures and Tables

**Table 1:** Experimental carts.

Cart Condition	Tire Type	Number of Carts Tested
Brand-new	Pneumatic	2
Brand-new	Solid	2
One-year-old (no noticeable structural damage)	Pneumatic	5
One-year-old (no noticeable structural damage)	Solid	5
Five-year-old (contaminated tires, broken/deformed bearings, warped axles, and loose turntables)	Solid (three years old)	2
Five-year-old (curved axles and loose turntables)	Solid (brand-new)	2

**Table 2:** Statistical results of Experiment 1a: Regression coefficients (β), robust standard errors (s.e.), and *p*-values from the final models, featuring independent variables: cart condition (brand-new as reference category) and cart load (243 kg as baseline) and dependent variables: ROF, CP, and ROT. Bold fonts indicate *p*-values are less than our significance level 0.05.

Effect	Required Operating Force, ROF (N)			Capability Percentage, CP (%)			Percentage Change in Required Operating Time, ROT (%)		
	β	s.e.	*p*-Value	β	s.e.	*p*-Value	β	s.e.	*p*-Value
Intercept	114	3.51	**<0.001**	83.6	1.95	**<0.001**	989	2.02	**<0.001**
Cart condition									
*One-year-old* vs. *brand-new*	5.76	4.61	0.215	−3.02	2.70	0.266	17.1	2.04	**<0.001**
*Five-year-old* vs. *brand-new*	65.5	4.61	**<0.001**	−30.6	3.52	**<0.001**	35.4	3.08	**<0.001**
*Five-year-old after tire replacement* vs. *brand-new*	27.1	5.98	**<0.001**	−13.4	2.21	**<0.001**	18.0	3.08	**<0.001**
Cart load (kg)	0.487	0.025	**<0.001**	−0.289	0.013	**<0.001**	0.046	0.008	**<0.001**

**Table 3:** Statistical results of Experiment 1b: Regression coefficients (β), robust standard errors (s.e.), and *p*-values from the final models, featuring independent variables: cart condition (brand-new as baseline), tire type (pneumatic tire as baseline), and cart load (243 kg as baseline), an interaction term between the tire type and cart load, and dependent variables: ROF, CP, and ROT. Bold fonts indicate *p*-values are less than our significance level 0.05.

Effect	Required Operating Force, ROF (N)			Capability Percentage, CP (%)			Percentage Change in Required Operating Time, ROT (%)		
	β	s.e.	*p*-Value	β	s.e.	*p*-Value	β	s.e.	*p*-Value
Intercept	92.6	3.0	**<0.001**	98.1	1.88	**<0.001**	1029	2.01	**<0.001**
Cart condition *(One-year-old* vs. *brand-new)*	11.7	2.79	**<0.001**	−7.30	1.82	**<0.001**	15.6	1.82	**<0.001**
*Tire type (solid* vs. *pneumatic)*	22.0	3.55	**<0.001**	−11.9	2.12	**<0.001**	−0.75	1.64	0.645
Cart load (kg)	0.326	0.023	**<0.001**	−0.201	0.017	**<0.001**	0.036	0.009	**<0.001**
Tire type × cart load	0.173	0.031	**<0.001**	−0.115	0.020	**<0.001**	-	-	**-**

**Table 4:** Statistical results of Experiment 2a: Regression coefficients (β), robust standard errors (s.e.), and *p*-values from the final models, featuring four independent variables: tire type (pneumatic tire as baseline), obstacle height (1.9 cm as baseline), space constraint (open space as baseline), and cart load (16 kg as baseline), interaction terms between the obstacle height and space constraint and between the cart load and space constraint, and finally dependent variables (ROF, CP, and ROT). Bold fonts indicate *p*-values are less than our significance level 0.05.

Effect	Required Operating Force, ROF (N)			Capability Percentage, CP (%)			Percentage Change in Required Operating Time, ROT (%)		
	β	s.e.	*p*-Value	β	s.e.	*p*-Value	β	s.e.	*p*-Value
Intercept	29.8	7.44	**0.008**	110	1.73	**<0.001**	353	2.67	**<0.001**
Tire type (solid vs. pneumatic)	60.5	5.91	**<0.001**	−14.7	1.38	**<0.001**	2.38	1.87	**0.003**
Obstacle height (cm)	25.0	2.23	**<0.001**	0.996	0.523	0.057	−1.83	0.612	0.204
Space constraint									
*Moderate* vs. *open space*	46.8	10.4	**<0.001**	0.744	2.56	0.772	−75.5	2.21	**<0.001**
*Extreme* vs. *open space*	190	15.2	**<0.001**	−45.0	4.68	**<0.001**	−88.6	2.36	**<0.001**
Cart load (kg)	0.546	0.022	**<0.001**	−0.210	0.005	**<0.001**	0.071	0.006	**<0.001**
Obstacle height × space constraint									
*Obstacle height ×* *Moderate* vs. *open space*	2.03	4.59	0.569	−3.77	0.486	**<0.001**	**-**	**-**	**-**
*Obstacle height ×* *Extreme* vs. *open space*	76.8	5.99	**<0.001**	−14.2	1.21	**<0.001**	**-**	**-**	**-**
Cart load × space constraint									
*Cart load ×* *Moderate* vs. *open space*	0.386	0.036	**<0.001**	−0.028	0.007	**<0.001**	**-**	**-**	**-**
*Cart load ×* *Extreme* vs. *open space*	0.845	0.057	**<0.001**	0.072	0.012	**<0.001**	**-**	**-**	**-**

**Table 5:** Statistical results of Experiment 2b: Regression coefficients (β), robust standard errors (s.e.), and *p*-values from final regression models, featuring four independent variables: tire type (pneumatic tire as baseline), ramp slope (4° as baseline), space constraint (open space as baseline), and cart load (16 kg as baseline), an interaction term between the ramp slope and cart load, and dependent variables: ROF, CP, and ROT. Bold fonts indicate *p*-values are less than our significance level 0.05.

Effect	Required Operating Force, ROF (N)			Capability Percentage, CP (%)			Percentage Change in Required Operating Time, ROT (%)		
	β	s.e.	*p*-Value	β	s.e.	*p*-Value	β	s.e.	*p*-Value
Intercept	37.5	2.54	**<0.001**	119	1.46	**<0.001**	275	1.95	**<0.001**
Tire type (solid vs. pneumatic)	31.5	1.91	**<0.001**	−6.96	0.848	**<0.001**	4.24	1.29	**0.0013**
Ramp slope (°)	10.3	0.385	**<0.001**	−2.29	0.217	**<0.001**	2.19	0.335	**<0.001**
Space constraint									
*Moderate* vs. *open space*	19.2	2.31	**<0.001**	−3.92	0.988	**<0.001**	6.28	1.56	**<0.001**
*Extreme* vs. *open space*	41.5	2.27	**<0.001**	−8.76	1.05	**<0.001**	11.9	1.56	**<0.001**
Cart load (kg)	0.756	0.009	**<0.001**	−0.196	0.004	**<0.001**	0.098	0.006	**<0.001**
Ramp slope × cart load	0.123	0.003	**<0.001**	−0.030	0.001	**<0.001**	0.015	0.002	**<0.001**

## Data Availability

The data presented in this study are available on request from the corresponding author.
